# Colonic Varices as an Unsettling Cause of Lower Gastrointestinal Bleeding

**DOI:** 10.7759/cureus.60490

**Published:** 2024-05-17

**Authors:** Jayasree Ravilla, Du Doantrang

**Affiliations:** 1 Internal Medicine, Monmouth Medical Center, Long Branch, USA

**Keywords:** tips, cirrhosis, gastrointestinal bleeding, varices, colon

## Abstract

Colonic varicose veins are very rare and are usually discovered incidentally during colonoscopy or when complications occur, such as lower gastrointestinal (GI) bleeding. The primary cause of colonic varices is usually portal hypertension secondary to liver disease or very rarely due to pancreatic disease (e.g., pancreatic adenocarcinoma). Varicose veins secondary to cirrhosis are often seen in the upper GI tract but rarely in the lower GI tract. Here, we report a 54-year-old woman who presented with colonic varices due to decompensated alcoholic cirrhosis. The main intention of this case report was to raise awareness of the possibility of developing colonic varices from liver cirrhosis and to promptly identify and manage its side effects due to the major complication which is lower GI bleeding.

## Introduction

Colonic varices are a rare cause of gastrointestinal (GI) bleeding. Colon varices are usually caused by liver cirrhosis and lesser etiological causes include congestive heart failure, splenic vein and mesenteric vein thrombosis, and rarely due to idiopathic factors. We present a fatal case of colonic varices secondary to liver cirrhosis [1-3]. Dilated, circuitous portosystemic collateral veins that are situated outside of the gastro-esophageal region are referred to as ‘ectopic varices’. It is estimated that only 3.4 % of the patients who develop portal hypertension secondary to liver cirrhosis develop colonic varices [4,5]. In this report, we present an unfortunate case of fatal colonic varices secondary to alcoholic liver cirrhosis.

## Case presentation

A 54-year-old woman with a history of alcoholic cirrhosis (graded child-pugh class C with a score of 15) and grade III esophageal varices (revised Japanese classification) presented to the emergency department complaining of bright red blood per rectum for one day. Additionally, she reported symptoms of dizziness and fatigue. She had a history of chronic alcoholism that spans nearly 30 years. She denied any hematemesis, any melena, change in her stool, recent antibiotic use, recent travel history, weight changes, and any illicit or recreational drug use. Her home medications included Lasix 40 mg and spironolactone 100 mg orally daily for ascites, which she complies without any side effects. She was worked up for liver transplant but was deferred transplantation due to the elevated phosphatidylethanol (Peth) value at > 200 ng/ml a week prior to admission.

**Figure 1:**
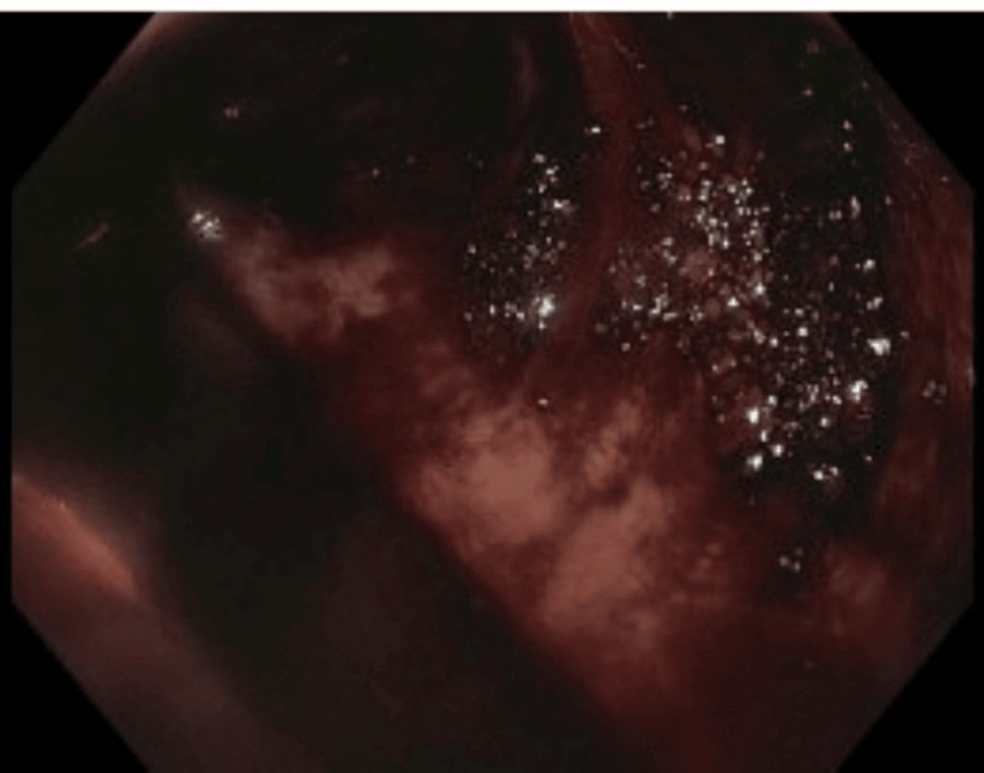
Colonoscopy image Emergent colonoscopy showing poor bowel preparation and colonic lumen obscured with blood.

Her initial vitals were stable and labs revealed a drop in her hemoglobin (Hb) from 11 g/dl (baseline) to 9 g/dl. Physical examination revealed a distended abdomen with signs of ascites. On physical examination, she had no signs of encephalopathy, but orthostatic vital signs were positive. A comprehensive metabolic panel (CMP) showed an albumin level of 2.8 g/dL. Esophagogastroduodenoscopy was performed which revealed grade III esophageal varices, grade A esophagitis (Los Angeles Classification), portal hypertensive gastropathy, and normal duodenum. However, flexible colonoscopy was deferred due to poor prep and large blood of blood in the rectum and was scheduled for a later date (Figure 1). 

She was transferred to the Intensive Care Unit (ICU) due to persistent orthostatic vitals and for closer hemodynamic monitoring (Table 1).

**Table 1:** Lab values of the patient Basic labs pertinent to the case.

Labs	Hour 0	Hour 6
White blood cell (4,000–11,000 cells/mm^3^)	4530 cells/mm^3^	9000 cells/mm^3^
Hemoglobin (11–15.1 g/dL)	10.8 g/dL	6.1 g/dL
Hematocrit (33.1%–44.5%)	30%	21.2%
Platelet (150,000–400,000 cells/mm^3^)	200,000 cells/mm^3^	190,000 cells/mm^3^
Alkaline phosphatase (32–91 U/L)	40 U/L	80U/L
Aspartate aminotransferase (15–41 U/L)	20 U/L	22 U/L
Alanine aminotransferase (7–52 U/L)	45 U/L	40 U/L
Total bilirubin (0.3–1 mg/dL)	1 mg/dL	0.9 mg/dL
International normalized ratio (0.9–1.1)	1.5	1.9
Blood urea nitrogen (6–20 mg/dL)	29 mg/dL	34 mg/dL
Creatinine (0.44–1.03 mg/dL)	1 mg/dL	1.03 mg/dL
Serum albumin (3.5–5.1 g/dL)	3.2 g/dL	3 g/dL
Urine drug screen	negative	
Blood alcohol (<10 mg/dL)	normal	

**Figure 2:**
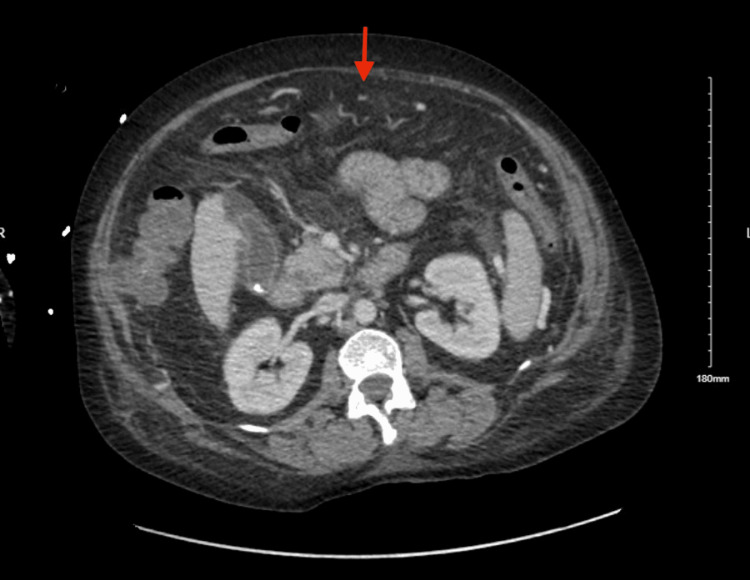
CT enterography There was active extravasation of blood in the right colon.

A computed tomography (CT) scan with intravenous contrast of the abdomen and pelvis revealed liver cirrhosis, patent portal vein, splenic artery aneurysm, and multiple varices of the right colon, which were new findings compared with the abdominal scan she had performed a year earlier. It did not show any active extravasation. On the same day, within six hours of presentation to the emergency department, the patient developed hemodynamic instability with hypotension due to massive lower GI bleeding. Vital signs revealed a blood pressure of 60/50 mm Hg. The massive blood transfusion protocol was initiated and she required multiple blood transfusions, but subsequent CT enterography showed colonic varices but no extravasation (Figure 2).

**Figure 3:**
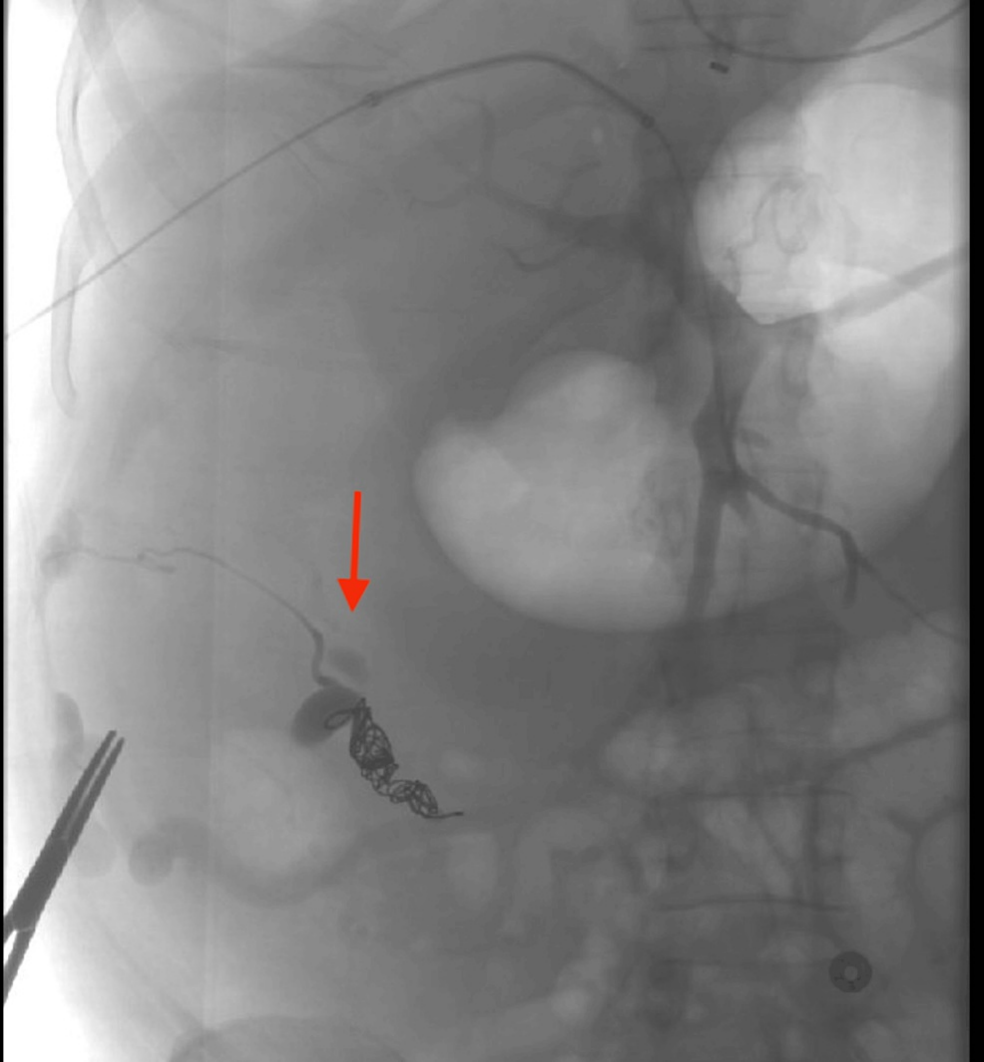
IR-guided superior mesenteric vein coiling

Interventional radiology was consulted due to emergent situation and CT mesenteric angiography showed pancolonic varices but no active bleeding site was identified. As no active source of bleeding was identified, a transjugular intrahepatic portosystemic shunt (TIPS) was planned. During the procedure, a right colonic variceal connection was discovered near the superior mesenteric vein and successfully embolized with a coil (Figure 3). 

Although the portal system was patented, the TIPS could not be created due to technical difficulties in accessing the system via the right hepatic vein. She became very hemodynamically unstable with blood pressure (50/32 mm Hg) not responding to blood transfusions and vasopressors. Unfortunately, she suffered cardiac arrest due to hypovolemic shock and died within a day of arrival.

## Discussion

GI varices of the distal colon which can present as isolated colonic varices have not been fully explored. In patients with cirrhosis, upper GI varices, i.e., esophageal varices are seen in 50 % of the cases. The presence of upper GI varices in the esophagus or stomach appears to have nil effect on the occurrence of these lesions [6,7]. The risk of hemorrhage significantly increases with the prevalence of colonic varices at a rate of 12 % annually [6,7]. Though the common etiology is primarily portal hypertension, it can occur in idiopathic cases. GI bleeding ranging from mild to fatal cases were found in the literature [8]. Here, we focus on this case of pancolonic varices presenting with life-threatening rectal bleeding.

Varices in the GI tract arise from portal hypertension secondary to increased intrahepatic vascular resistance and elevated blood flow through the portal and collateral venous systems [4]. The sites commonly involved include jejunum or ileum (18%), duodenum (17%), rectum (8%), and peritoneum (9%). Rectal and cecal varices are commonly seen during colonoscopy in the lower GI tract but isolated colonic varices are extremely rare [9]. The pathophysiology of cirrhosis includes the advancement of portal hypertension leading to collateral vessel formation and arterial vasodilation resulting in significantly elevated blood flow in portal circulation. This event creates a hyperdynamic circulatory state leading to the formation of varices and although esophageal varices are the common variant, it can also lead to ectopic lesions like pancolonic varices. It is found that nitric oxide (NO) plays a vital role in increased vasodilation thus leading to portosystemic collateral vessels in response to angiogenesis [9,10].

The majority of patients are asymptomatic during the presentation but few can be present with detrimental lower GI bleeding. CT angiography or mesenteric angiography is the most accurate diagnostic tool, with identification of varices during the venous phase [4]. Colonoscopy is the diagnostic option, although the findings are discovered incidentally during the procedure. During the endoscopy, colonic varices are identified by dilated, tortuous vascular tracts with a bluish tinge on the mucosal surface. Colonoscopy also helps to know the extent of the disease, thus helping the treatment course [10,11]. Our patient was diagnosed appropriately with colonic varices through CT enterography and colonoscopy with appropriate diagnostic modules.

In emergency situations, some successful treatment options include TIPS, balloon retrograde transvenous occlusion (BRTO), and venous coil embolization. Usual interventions like banding of varices and sclerotherapy are used when varices are limited to the rectum, but they are generally avoided in proximal involvement due to the risk of perforation. Alternatively, beta-blockers, which are commonly used for medical management of esophageal varices, can be used but most of the colonic varices are discovered with the complications of active GI bleeding [12]. In many cases, TIPS proved to be an effective way to stabilize patients with active bleeding. In patients with failed TIPS, venous coil embolization of colonic varices with tissue propylene injection can be performed. Complications of this procedure like vessel perforation, migration of coil, necrosis, and embolization of the non-target lesion should be considered [3,4]. In very few documented cases, BRTO, an endovascular procedure, is performed with some success. Subtotal colectomy has also been reported as the treatment option but regrettably due to the rarity of cases, there are no established treatment guidelines for management of colonic varices [13].

## Conclusions

The prevalence of colonic varices is difficult to establish because this condition remains subclinical for most of the time. Unfortunately, due to the rarity of colonic varices, there are currently no adequate treatment guidelines. Although surgical intervention is the next treatment choice after IR intervention and banding through colonoscopy, it is often challenging due to the coagulopathy caused by cirrhosis. The aim of the treatment is to decrease portal hypertension, which is the primary cause of bleeding colonic varices. Delays in diagnosis and treatment can lead to serious consequences, including death.
